# Septic arthritis of the shoulder due to *Ureaplasma urealyticum* after emergency caesarean section: a case report

**DOI:** 10.1186/s12879-020-05497-3

**Published:** 2020-10-17

**Authors:** Jaad Mahlouly, Loic Lhopitallier, Véronique Suttels, Linda Mueller, Diane Wernly, Olivier Borens, Sylvain Steinmetz

**Affiliations:** 1 Department of Orthopaedics and Traumatology, Lausanne University Hospital (CHUV), Rue du Bugnon 46, 1011 Lausanne, Switzerland; 2 Department of Infectious Diseases, Lausanne University Hospital (CHUV), Lausanne, Switzerland; 3 Institute of Microbiology, University of Lausanne, Lausanne University Hospital (CHUV), Lausanne, Switzerland

**Keywords:** *Ureaplasma urealyticum*, Septic arthritis, Caesarean section, Post-partum infection, Case report

## Abstract

**Background:**

*Ureaplasma urealyticum* is an intra-cellular bacterium frequently found colonizing the genital tract. Known complications include localized infections, which can result in premature deliveries. Septic arthritis due to *U. urealyticum* in healthy patients is exceptionally rare, although opportunistic septic arthritis in agammaglobulinemic patients have been reported. However, there are no reports of septic arthritis due to *U. urealyticum* following caesarean section or in the post-partum period.

**Case presentation:**

A 38-year-old immunocompetent woman presented with severe right shoulder pain, 1 month following emergency caesarean section at 26 weeks of gestation for pre-eclampsia and spontaneous placental disruption with an uncomplicated post-operative recovery.

Our suspicion of septic arthritis was confirmed with abundant pus following arthrotomy by a delto-pectoral approach. Awaiting culture results, empirical antibiotic treatment with intravenous amoxicilline and clavulanic acid was initiated. In spite of sterile cultures, clinical evolution was unfavorable with persistent pain, inflammation and purulent drainage, requiring two additional surgical débridement and lavage procedures.

The 16S ribosomal RNA PCR of the purulent liquid was positive for *U. urealyticum* at 2.95 × 10^6^ copies/ml, specific cultures inoculated a posteriori were positive for *U. urealyticum*. Levofloxacin and azithromycine antibiotherapy was initiated. Susceptibility testing showed an intermediate sensibility to ciprofloxacin and clarithromycin. The strain was susceptible to doxycycline. Following cessation of breastfeeding, we started antibiotic treatment with doxycycline for 4 weeks. The subsequent course was favorable with an excellent functional and biological outcome.

**Conclusions:**

We report the first case of septic arthritis due to *U. urealyticum* after caesarean section. We hypothesize that the breach of the genital mucosal barrier during the caesarean section led to hematogenous spread resulting in purulent septic arthritis. The initial beta-lactam based antibiotic treatment, initiated for a purulent arthritis, did not provide coverage for cell wall deficient organisms. Detection of 16S rRNA allowed for a correct microbiological diagnosis in a patient with an unexpected clinical course.

## Background

*Ureaplasma urealyticum*, a short intra- and extracellular rod lacking a cell wall, frequently colonizes the genital human tract. In the genital tract of sexually active healthy women, prevalence estimates range from 38 to 75% [1, 2]. This germ belongs to the genital mycoplasmas, containing *Ureaplasma* spp*.* and *Mycoplasma* spp., seven of which are referenced in humans on mucous membranes: *U. urealyticum*, *U. parvum*, *M. pneumonia*, *M. ferentans*, *M. penetrans*, *M. genitalium* and *M. hominis* [1, 3]. They colonize mucosal surfaces and are mostly non-pathogenic, except for *M. pneumoniae,* which causes respiratory tract infections [1]. However, they all have the potential to invade tissues and cause opportunistic infections. To date, their exact pathogenicity remains to be determined.

The genus *Ureaplasma* is divided into two commensal species, *U. urealyticum* and *U. parvum*. *U. urealyticum* has been more frequently implicated in opportunistic infections in antibody deficient patients [3]. In the absence of antibodies, neutrophils phagocyte these bacteria, however they remain viable and are likely to proliferate [4].

Genital tract infection and premature birth have been associated to infection with *U. urealyticum* [5].. Some twenty cases of patients with septic arthritis due to *U. urealyticum* have been described in the literature and in all but two cases antibody deficiencies were identified [6–8].

Bone and joint infections can occur in the postpartum period, such as sacroiliitis by contiguous spread. Septic arthritis of a limb is rarer, and half a dozen described cases have revealed germs such as *S. aureus*, *M. hominis* or *S. agalactiae* [9–11].. However, there are no reports of septic arthritis due to *U. urealyticum* after caesarean section in the postpartum period in an immunocompetent patient.

## Case presentation

In December 2019, a right-handed 38-year-old woman presented to the emergency department with increasing right shoulder pain and raised inflammatory parameters. Her past medical history was remarkable for paraplegia due to poliomyelitis in childhood and a residual epilepsy due to the sequelae of a subarachnoid hemorrhage. Apart from these conditions, for which she was wheelchair-bound, this patient was in good general health with a previous non-pathologic and well-functioning right shoulder.

One month prior to her admission with shoulder pain, she was involved in a car accident as a result of a tonic-clonic seizure. Upon admission, she developed hypertension with associated proteinuria and was diagnosed with a 26-week gestation previously unknown pregnancy and pre-eclampsia. She was previously nulliparous. At day six of hospitalization, the patient presented a spontaneous placental abruption and underwent an emergency caesarean section with a simple post-operative evolution.

Physical exam of her right upper extremity revealed a swollen, warm and painful right shoulder on palpation and mobilization without neurovascular disorder. On general examination, she had moderate asthenia without disorientation. The vital signs were: Temperature of 37.2 °C, heart rate at 104 beats per minute, blood pressure of 148/95 mmHg, respiratory rate at 23 per minute and an oxygen saturation within the norm (SpO2 95% breathing ambient air).

Hemoglobin was 101 G/L, leukocytes 9.8 G/L, thrombocytes 305 G/L and C-reactive protein was increased to 271 mg/L. Blood cultures were sent for culture.

**Fig. 1:**
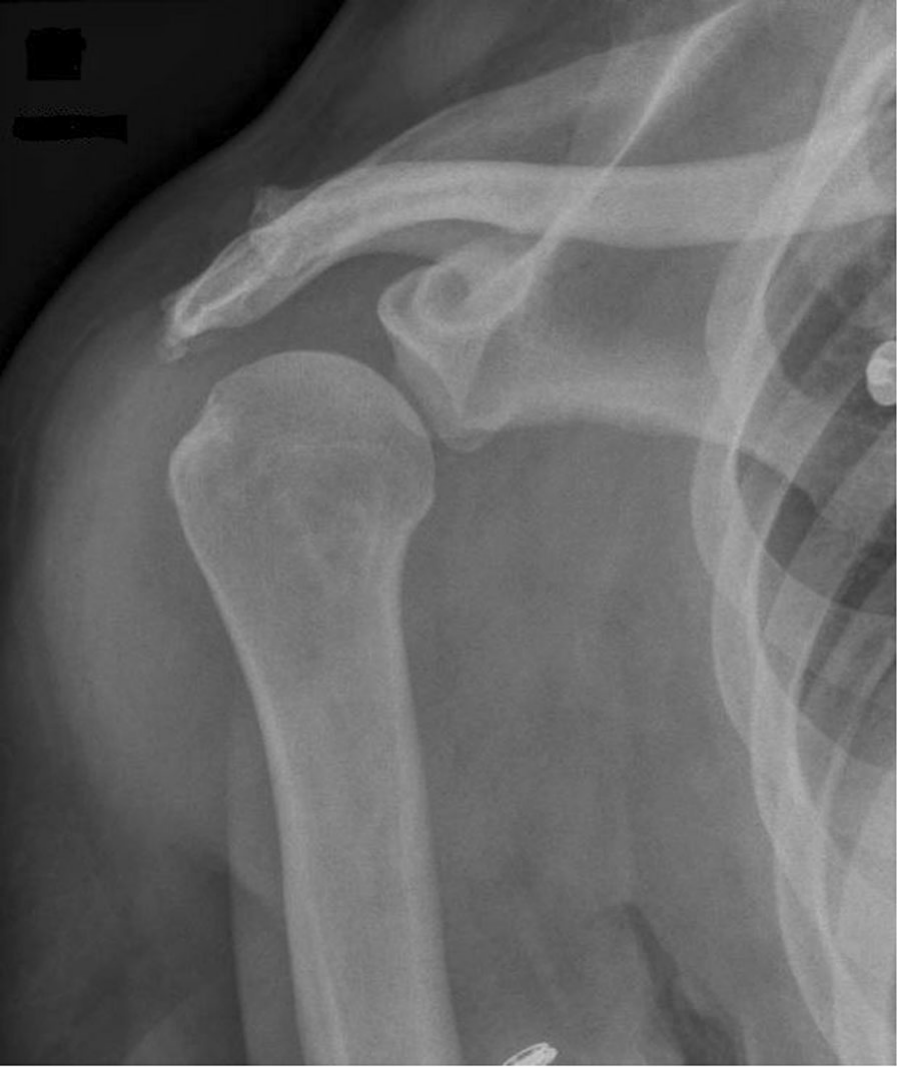
Pre-operative AP right shoulder Xray without degenerative signs

Suspecting right shoulder septic arthritis without any degenerative sign on X-ray (Fig. 1), she underwent open surgical debridement, synovectomy and 9 L saline solution lavage of the right shoulder by a delto-pectoral approach with subscapularis tenotomy. Abundant pus at arthrotomy was observed with the observation of a stage 3 equivalent according to Gächter’s classification [12].

Awaiting culture results, empirical antibiotic treatment with intravenous 2,2 g 3td amoxicilline and clavulanic acid was initiated.

**Fig. 2:**
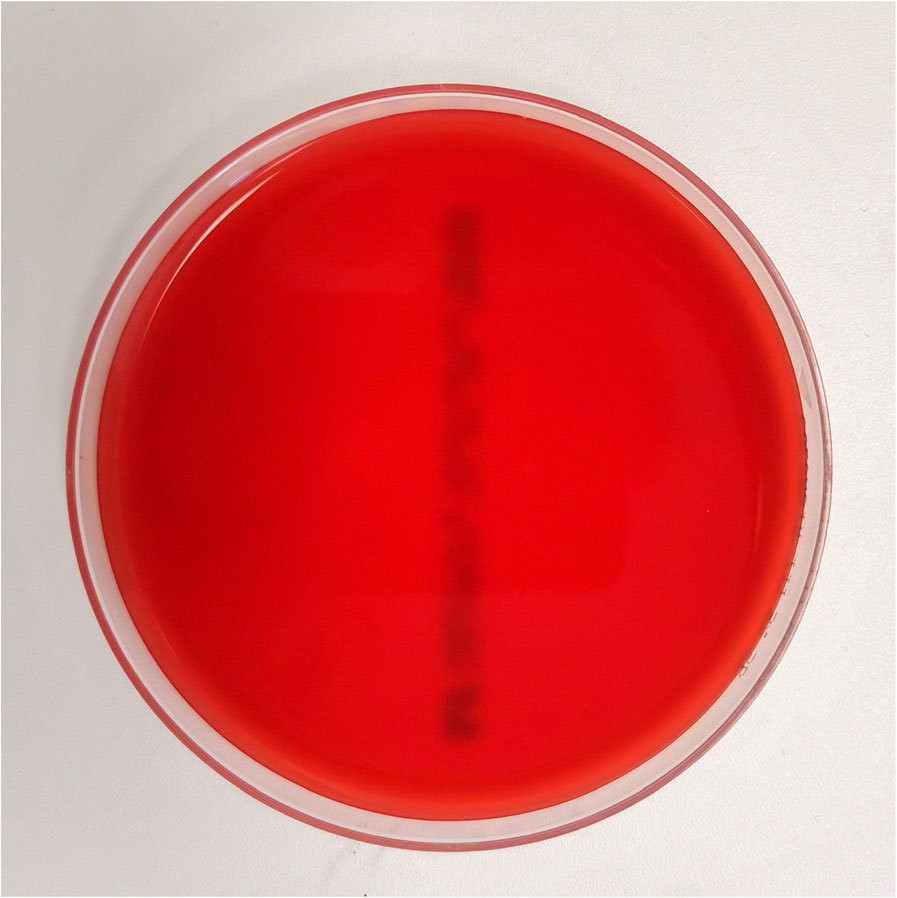
Classic blood agar with absence of U. urealyticum growth

Whilst the blood cultures and intra-operative cultures remained sterile (Fig. 2) without any crystals positivity on microscopic analysis, the clinical evolution was unfavorable with persistent pain, inflammation and purulent drainage. The patient required two additional surgical debridements and lavages on days 4 and day 7 after the first surgery. Both surgical interventions revealed persistent amounts of purulent and fibrous material despite the previous procedures and the empirical antibiotic treatment.

**Fig. 3:**
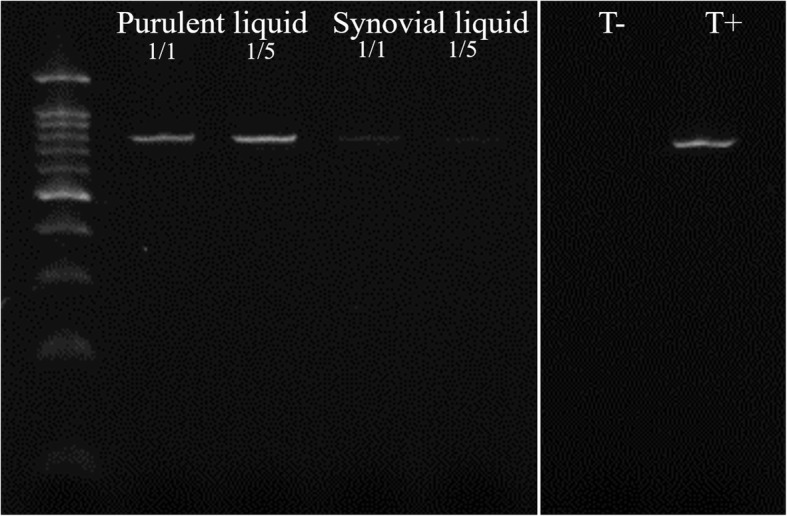
PCR amplification identifiing U. urealyticum (Purulent liquid and Synovial liquid 1/1 and 1/5 are different dilutions of samples. T- and T+ are negative and positive controls)

**Fig. 4:**
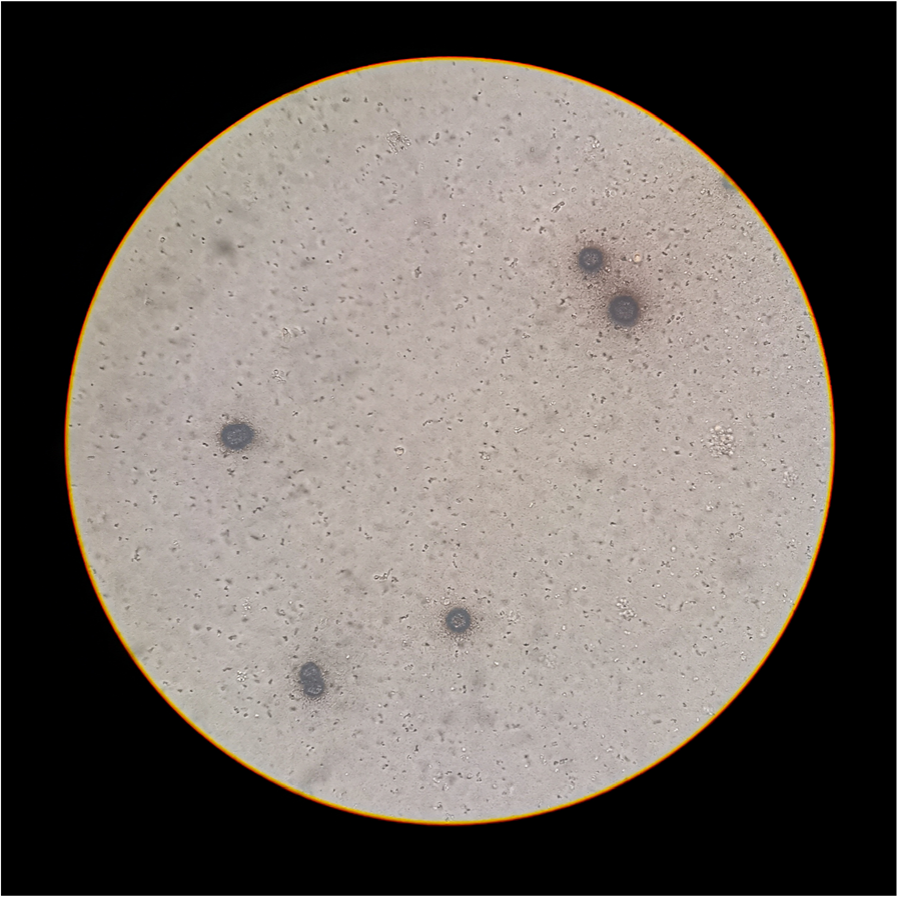
Microscopic view of characteristic U. urealyticum colonies on rich media supplemented with yeast extract and urea (10x)

The 16S ribosomal RNA PCR performed on the purulent intra-operative liquid revealed *U. urealyticum* at 2.95 × 10^6^ copies/ml (Fig. 3), positive specific cultures inoculated a posteriori confirmed this finding (Fig. 4).

Cervical smear showed no simultaneous *U. urealyticum* genital infection. Fresh placental tissue was not available for further testing, but there was no evident macroscopic chorio-amnionitis.

**Fig. 5:**
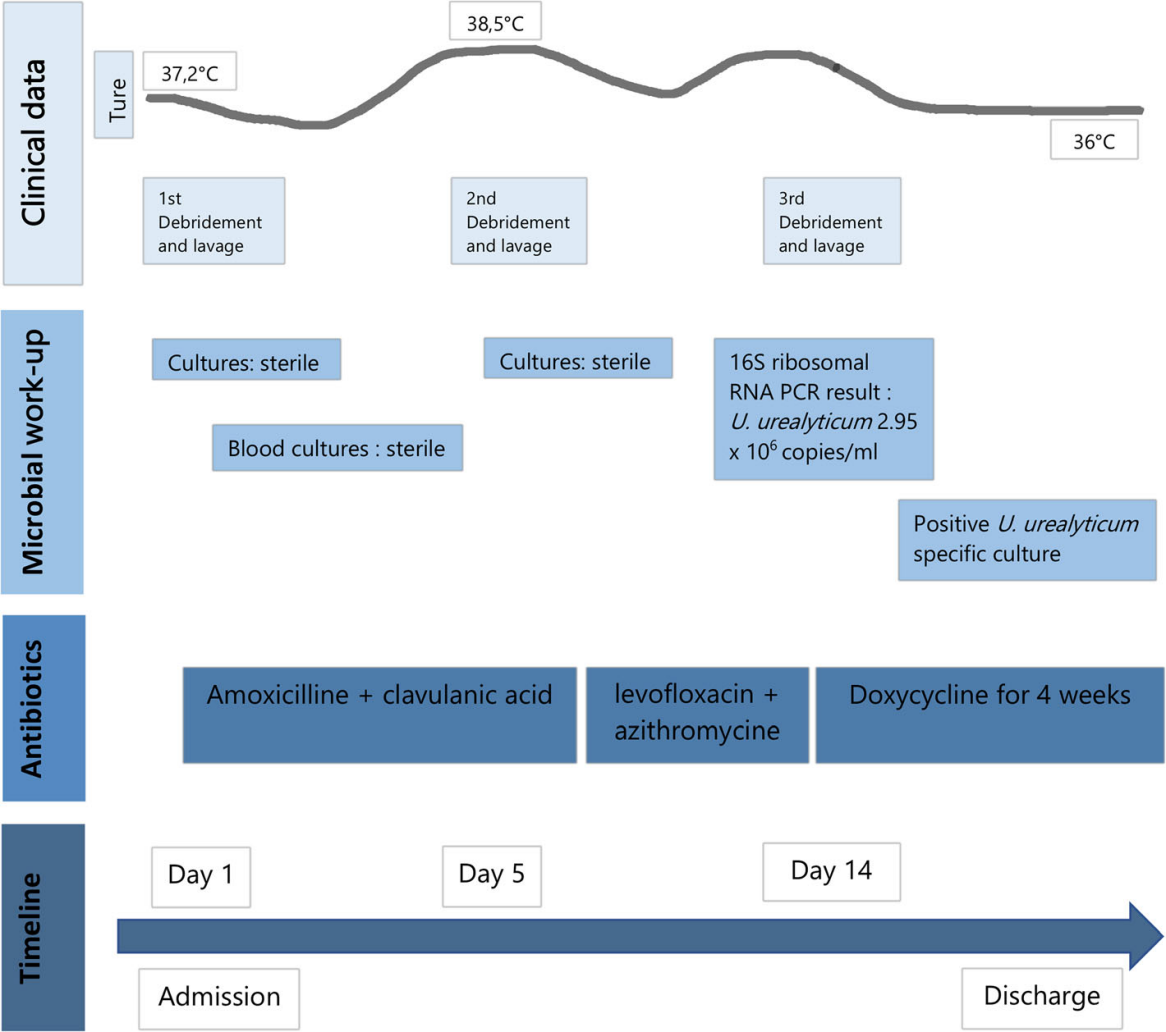
Timeline of clinical events and work-up

Upon receiving the microbiological documentation, a bi-therapy of levofloxacin and azithromycine was initiated. Susceptibility testing showed an intermediate sensibility to ciprofloxacin and clarithromycin. The strain was susceptible to doxycycline. Following cessation of breastfeeding, we started treatment with doxycycline for 4 weeks according to the guidelines of our institution and on the basis of the low initial therapeutic response [13]. Early mobilization was started with our physiotherapist (Fig. 5). She showed a favorable course with an excellent biological and functional outcome with a post-surgical DASH score of 5,8 at 3 months follow-up [14]. As a reminder, a DASH score of 0 represents no disability and a score of 100 represents severe disability after surgery. Concerning the father of the infant, no other specific considerations were taken related to this infection. The infant presented good clinical evolution.

## Discussion and conclusions

Arthralgias during pregnancy and in the postpartum period are common, moreover our patient did not present with fever on admission [15]. These common arthralgias may be due to hormonal changes and changes in joint ligament laxity, a condition more common in the lower limbs [9]. It is therefore important to perform a laboratory work-up to differentiate these arthralgias from inflammatory or infectious pathology.

*U. urealyticum* is frequently found in the genital tract and normally elicits a response from the humoral immune system [1, 4]. Deficits in this type of response are a risk factor for septic arthritis [8]. In the absence of antibodies, these organisms are phagocytized by neutrophils but are not degraded and remain viable and able to proliferate [4]. To date and to our knowledge, only two cases of *U. urealyticum* septic arthritis have been reported in healthy patients, both with involvement of the knee [6, 7].

Concerning septic arthritis in the postpartum period, there are to our knowledge only eight cases described in the limb joints: wrist, hip, knee and only one shoulder. They all involve germs who were located in the genital tract (*S. aureus*, *M. hominis* or group B streptococci) and some of them have occurred after caesarean section procedures [9–11]. Involvement of the sacroiliac joint or the pubis is more frequent and probably due to contamination by continuous spread [16].

We believe that the breach of the genital mucosal barrier during the caesarean section led to hematogenous spread inoculating the shoulder and causing purulent septic arthritis. Antibiotic prophylaxis prior to genital surgery is important and can reduce the incidence of perioperative infections, but it cannot eradicate them, especially since it does not cover all the different types of pathogens [17, 18]. The atypical presentation at 3 weeks post-surgery could be explained by the post-partum maternal immune boost, with increased innate and specific immune deficiencies, revealing a previously clinically silent infection [19].

Septic arthritis of native shoulder can have serious local consequences such as rapid joint degeneration or loss of function or cause systemic implication such as septic shock [9, 20]. Half of the patients with acute septic arthritis of native shoulder do not regain their previous level of activity after such a condition [21]. Treating the causative agent radically with surgery in conjunction with targeted adjuvant antibiotic therapy is essential to preserve function of the shoulder and reduces systemic complications [22]. All the more so in a patient who is paraplegic and right-handed.

*Mycoplasma* spp*.* and *Ureaplasma* spp. should be included in the microbiological differential diagnosis of the etiology of culture sterile purulent arthritis in the postpartum period.

Beta-lactam antibiotic treatment, initiated empirically for a purulent arthritis, does not provide coverage for cell wall deficient organisms. Failure of beta-lactam antibiotics in purulent arthritis raises a suspicion of resistant or atypical causal pathogens such as mycobacteria, fungi or *Mycoplasma* spp. Indeed, beta-lactams have no effect on *Mycoplasma* spp*.* lacking precisely the cell-wall targeted by these antibiotics. When confronting purulent arthritis with repeated sterile cultures, bacterial identification using PCR detection of 16S ribosomal RNA can sometimes allow for a correct microbiological diagnosis given its suboptimal sensitivity of about 58% and specificity of 85%. A positive test result can be validated a posteriori using specific PCR probes and culture media [23, 24].

In conclusion, we described the first case of septic arthritis due to *U. urealyticum* after genital surgery or in the post-partum period. We note all the more the fact that this is, to our knowledge, the third septic arthritis and specifically the first shoulder septic arthritis due to *U. urealyticum* in an immunocompetent host. We hypothesize that genital intervention led to hematogenic spread and resulting in purulent septic arthritis.

When facing an adverse clinical course of purulent arthritis in spite of multiple surgical débridements and sterile cultures in the post-partum period, we advise additional testing by 16S rRNA PCR to look for atypical bacteria, such as *U. urealyticum* to improve the targeting of antimicrobial treatment.

## Data Availability

Whole medical history, vital signs and laboratory values are available in the patient computerized medical file of University Hospital of Lausanne, Switzerland.

## Abbreviations

DASH score: The disabilities of the arm, shoulder and hand (DASH) questionnaire
*Mycoplasma* spp.: Species of the genus *Mycoplasma*
PCR: Polymerase chain reaction
rRNA: Ribosomal ribonucleic acid
*U. urealyticum*: *Ureaplasma urealyticum*
*Ureaplasma* spp*.*: Species of the genus *Ureaplasma*
